# Quantum Simulation of Pseudo-Hermitian-*φ*-Symmetric Two-Level Systems

**DOI:** 10.3390/e24070867

**Published:** 2022-06-24

**Authors:** Chao Zheng

**Affiliations:** Department of Physics, College of Science, North China University of Technology, Beijing 100144, China; czheng@ncut.edu.cn

**Keywords:** quantum simulation, linear combination of unitaries, non-Hermitian, pseudo-Hermitian

## Abstract

Non-Hermitian (NH) quantum theory has been attracting increased research interest due to its featured properties, novel phenomena, and links to open and dissipative systems. Typical NH systems include PT-symmetric systems, pseudo-Hermitian systems, and their anti-symmetric counterparts. In this work, we generalize the pseudo-Hermitian systems to their complex counterparts, which we call *pseudo-Hermitian-φ-symmetric* systems. This complex extension adds an extra degree of freedom to the original symmetry. On the one hand, it enlarges the non-Hermitian class relevant to pseudo-Hermiticity. On the other hand, the conventional pseudo-Hermitian systems can be understood better as a subgroup of this wider class. The well-defined inner product and pseudo-inner product are still valid. Since quantum simulation provides a strong method to investigate NH systems, we mainly investigate how to simulate this novel system in a Hermitian system using the linear combination of unitaries in the scheme of duality quantum computing. We illustrate in detail how to simulate a general *P*-pseudo-Hermitian-φ-symmetric two-level system. Duality quantum algorithms have been recently successfully applied to similar types of simulations, so we look forward to the implementation of available quantum devices.

## 1. Introduction

Hermitian quantum systems are well known since closed quantum systems were focused on at the birth of conventional quantum mechanics. However, open and dissipative quantum systems are more common than closed systems in the real physical world, and cannot be described by Hermitian quantum theory. Therefore, non-Hermitian (NH) quantum theory [1,2] is attracting increased research interest. On the one hand, it extends conventional quantum theory and closely relates to open and dissipative systems [3,4,5,6,7,8,9,10,11]. On the other hand, NH systems have many novel properties and applications.

Typical NH systems include the parity-time-reversal (PT) symmetric systems, pseudo-Hermitian (PH) systems, and their anti-symmetric counterparts. PT-symmetric NH quantum systems have been focused on and investigated heavily since 1998 [12,13,14,15]. One important reason for this is that, besides Hermitian systems, PT-symmetric systems also keep the eigenvalues of *H* real in the exact PT phase. Due to its significance in both theory and potential applications, PT-symmetric quantum physics is developing rapidly and being investigated thoroughly from different aspects in a variety of systems [16,17,18,19,20]. In recent years, quantum simulations of PT-symmetric systems have been carried out, for example, fast and slow evolutions in the quantum brachistochrone problem [21,22,23], a generalized PT two-level system [24,25,26,27], and a PT-arbitrary-phase-symmetric system [28]. P-pseudo-Hermiticitian Hamiltonians were found to have real spectra in some conditions [29,30,31,32,33,34], while a necessary and sufficient condition is given for the reality of the spectrum of NH Hamiltonians admitting a complete set of biorthonormal eigenvectors in [31]. Properties of pseudo-Hermitian systems and their relationships with PT-symmetric systems are often investigated and discussed [35,36,37,38,39,40,41]. In this work, we extend pseudo-Hermitian Hamiltonians to the complex domain for the first time by introducing a phase factor $e^{i\varphi }$.

Inspired by Feynman, quantum simulation provides an efficient way to investigate nature [42]. It has become a strong tool to simulate novel quantum systems and discover featured properties. After a detailed quantum circuit is designed, an effective Hamiltonian is constructed, and the time-evolution of a quantum system can be simulated. Plenty of Hermitian systems and relevant phenomena have been investigated via quantum simulation methods [43,44,45,46,47,48,49,50,51,52,53]. In addition, they can also be applied to investigate NH systems in an effective way [23,24,25,26,27,28]. For example, quantum simulation of a *P*-pseudo Hermitian two-level system and its anti-symmetric counterpart has been proposed [54,55,56,57,58,59,60,61,62,63,64,65,66], making it possible to investigate these two NH systems in small quantum devices.

In this work, we investigate quantum simulation of the generalized *pseudo-Hermitian-φ-symmetric (PH-φ)* system, using the linear combination of unitaries (LCU) in the scheme of duality quantum computing [67] and the unitary-expansion (UE) techniques [8,9]. We optimize the quantum circuit and calculate the success probability. Furthermore, we discuss the implementations in NMR and quantum optics systems, expecting experimental realizations in the near future.

## 2. Complex Generalization of Pseudo-Hermitian Symmetry

Pseudo-Hermitian (PH) Hamiltonians $H_{PH}$ satisfy $\eta^{-1}{H_{PH}}^{†}\eta =H_{PH}$, where η is a linear Hermitian automorphism (invertible transformation) on the Hilbert space [33]. For example, η can be the parity (P) operator. Since η is not unique for a given $H_{PH}$ [34], it is called an η-pseudo-Hermitian for a fixed η. Notice that different η values define different symmetries, though they are referred to as pseudo-Herimiticity in general. Similar to PT and anti-PT symmetry, an anti-symmetry of PH Hamiltonian has been introduced [35], and we call this a pseudo-Hermitian anti-symmetric (PHA) Hamiltonian $H_{PHA}$, if $\eta^{-1}{H_{PHA}}^{†}\eta =-H_{PHA}$. $H_{PH}$ and $H_{PHA}$ can be seen as the real and imaginary counterparts of each other, since the latter can be obtained by the former Hamiltonian times *i*, and vice versa.

Consider a non-Hermitian Hamiltonian $H_{\varphi }$, which satisfies

(1) $$\eta^{-1}{H_{\varphi }}^{†}\eta =e^{i\varphi }H_{\varphi }.$$

$H_{\varphi }$ can be obtained by a phase factor $e^{-i\frac{\varphi }{2}}$ times a relevant $H_{PH}$ (i.e., $H_{\varphi }=e^{-i\frac{\varphi }{2}}H_{PH}$). Therefore, $H_{\varphi }$ can be seen as a complex generalization of a conventional pseudo-Hermitian symmetry

(2) $$H_{\varphi }=e^{-i\frac{\varphi }{2}}H_{PH}=\cos\frac{\varphi }{2}H_{PH}-\sin\frac{\varphi }{2}\left(iH_{PH}\right).$$

$H_{\varphi }$ can also be seen as a combination of an $H_{PH}$ and a relevant $H_{PHA}=iH_{PH}$, which should have properties intermediate between them.

(3) $$H_{\varphi -PH}=\cos\frac{\varphi }{2}H_{PH}-\sin\frac{\varphi }{2}H_{PHA}.$$

Thus, we introduce one extra degree to the conventional pseudo-Hermitian symmetry, called $H_{\varphi }$, which is of η-*pseudo-Hermitian-arbitrary-phase* or *η-pseudo-Hermitian-φ* symmetry. Both the inner product and pseudo-inner product [34] of $H_{PH}$ are still well defined for $H_{\varphi }$. The relationships between PH, PH-anti, and PH-φ symmetry can be analogous to that of PT, anti-PT, and PT-arbitrary-phase symmetry, and can also be analogous to relationships between boson, fermion, and anyon. In fact, the relation in Equation (1) unifies the PH and PH-anti symmetries for φ=2kπ and φ=(2k+1)π (*k* is integral), respectively.

## 3. Quantum Simulation Using LCU by Duality Quantum Computing

We now propose how to simulate the time-evolution of a pseudo-Hermitian-φ- symmetric (PH-φ) system in a conventional unitary quantum computer. The time-evolutionary operator should be an implicit function of the PH-φ Hamiltonian $H_{\varphi }$, say $E\left(t\right)=E(H_{\varphi }\left(t\right))$. Given that $H_{\varphi }$ is not Hermitian, E(t) is not unitary and cannot be simulated directly by a quantum computer governed by conventional quantum mechanics, of which the time-evolution is unitary. However, E(t) can be extended to a summation of unitary operators. Therefore, we are able to simulate the non-unitary time-evolution of our PH-φ NH systems using LCU based on the duality quantum algorithm [67].

LCU and duality quantum computing were proposed in 2002 [67], and they developed rapidly [67,68,69,70,71,72,73], becoming some of the strongest tools for designing quantum algorithms [74]. Recently, we developed LCU to simulate NH systems [23,24,25,26,27,28,35,64,65], other novel systems, and time-dependent non-unitary operators [8].

Assume that the time-evolutionary operator can be extended to *m* terms as

(4) $$E\left(t\right)=\sum_{k=0}^{m-1}c_{k}U_{k},$$

where each $U_{k}$ is a unitary operator, and $c_{k}$ are complex UE parameters (k=0,…,m−1). The unitary expansion of E(t) is not unique, and we only show the schematic strategy to simulate the time-evolution of a general PH-φ non-Hermitian system in this section. We do not discuss details of how to extend the general non-unitary operator by our UE techniques, because this is a significant question and deserves to be investigated alone. However, it can save qubits and reduce the complexity of quantum simulation if the non-unitary operators can be expressed by fewer UE terms. We will illustrate in detail the quantum simulation of a *P*-pseudo-Hermitian-φ-symmetric two-level system in the next section.

**Figure 1:**
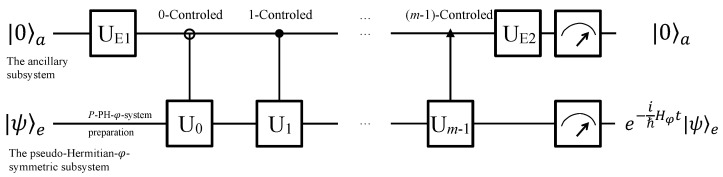
Schematic circuit for the quantum simulation of a PH-φ system based on LCU. The whole system consists of $n_{1}$ ancillary qubits and $n_{2}$ evolutionary qubits, and it will pass the quantum circuit from the left to the right. The system is initialized in ${|0\rangle }_{a}{|0\rangle }_{e}$ at first, and then the evolutionary qubits are prepared in arbitrary state ${|\psi \rangle }_{e}$ as demanded. After being operated by a unitary rotation $U_{E1}$, *m*-controlled operations (i.e., 0-controlled $U_{0}$, 1-controlled $U_{1}$, …, and (m−1)-controlled $U_{m-1}$), and a single-qudit rotation $U_{E2}$, the evolutionary subsystem will evolve as per Equation (4) if the ancillary subsystem is measured in state ${|0\rangle }_{a}$.

Quantum simulation of the time-evolution of a PH-φ system in Equation (4) can be achieved using either qudits or qubits as the quantum circuit, as shown in Figure 1. The whole system is composed of an ancillary subsystem *a* and an evolutionary subsystem *e*. The simulation can be achieved in either a qudit system or a qubit system. The dimensions of qudits or the total number of the qubits are decided by the dimensions *d* of the PH-φ non-Hermitian system and the number *m* of the UE terms in Equation (4). In detail, an *m*-dimensional qudit and a *d*-dimensional qudit are able to be the ancillary and evolutionary subsystems, respectively. If we simulate using qubits, the qubit numbers of the ancillary and evolutionary subsystems should not be less than $n_{1}=log_{2}m$ and $n_{2}=log_{2}d$, respectively. In general cases, for different dimensional NH systems, *m* has a maximum value of four [8,9]. In detail, the maximum of *m* is three in general and two in special cases for d=2 [8], while the maximum of *m* is four for higher dimensions [9]. At the beginning, the ancillary subsystem is initialized to a logic state ${|0\rangle }_{a}$, and the PH-φ non-Hermitian subsystem is initialized to an arbitrary state ${|\psi \rangle }_{e}$. In the middle part, the operator $U_{E1}$ is applied on the ancillary subsystem to assign the UE parameters, and the *k*-controlled gates (k=0,1,…,m−1) together with the operator $U_{E2}$ achieve the UE terms’ generation [8], constructing the PH-φ system. At the end of the circuit, quantum measurements will be performed on the ancillary system to complete the time-evolution governed by the PH-φ Hamiltonian in an indeterministic way when the ancillary qubits collapse into the logic state ${|0\rangle }_{a}$. In this case, the evolutionary subsystem will evolve as the PH-φ system requires. Otherwise, this simulation will be terminated and started over until success.

## 4. Quantum Simulation of P-Pseudo-Hermitian-φ-Symmetric Two-Level Systems

**Figure 2:**
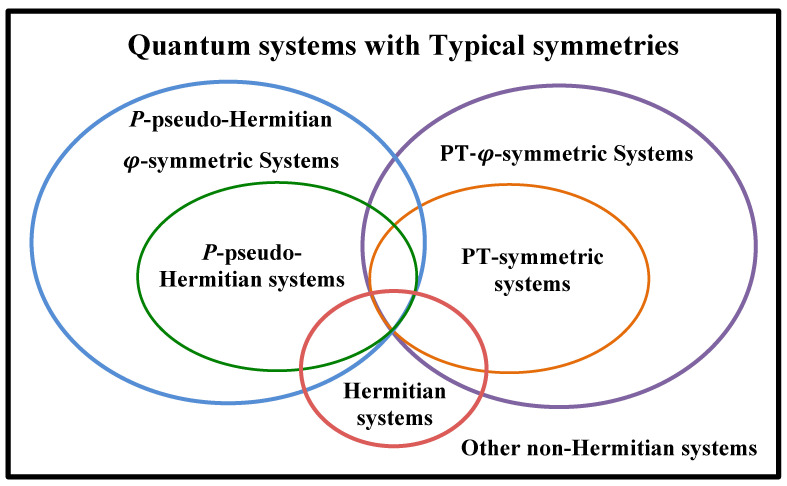
Sets of the *P*-pseudo-Hermitian-φ-symmetric, PT-φ-symmetric, *P*-pseudo-Hermitian, and Hermitian systems. The green ellipse of the *P*-PH set is in the blue ellipse of the general *P*-PH-φ set because the former can be seen as a special case where φ=2kπ (*k* is integral) of the latter. The *P*-PH anti-symmetric set is also a subset of the *P*-PH-φ set when φ=(2k+1)π (*k* is integral). The sets relevant to the *P*-pseudo-Hermiticity and the PT symmetry are different, though they have intersections with each other. Notice that the Hermitian sets are not in the *P*-PH-φ set only. Other NH sets may include various η-PH-φ sets (η is other than *P*), sets relevant to unknown symmetries, and so on.

We take a *P*-pseudo-Hermitian-φ-symmetric system as an example to illustrate how to simulate it using LCU and duality quantum algorithms. *P*-pseudo-Hermitian systems are one class of typical NH systems, and a series of theoretical investigations have been conducted on them. Recently, pseudo-Hermiticity was found to be able to protect unitary scattering [41]. *P*-PH systems have close relationships with PT-symmetric systems, though they are different. We have generalized the PT symmetry to PT-arbitrary-phase symmetry [28], which also has close relationships with *P*-PH-φ symmetry. We show the sets of the *P*-PH-φ, PT-φ-symmetric, *P*-PH, PT-symmetric, and Hermitian systems in Figure 2. They have intersections in the *P*-PH-related sets, PT-symmetry-related sets, and Hermitian set.

### 4.1. P-PH-φ Two-Level Systems

In the two-dimensional case, the most general form of a *P*-PH-φ Hamiltonian can be obtained by the relevant *P*-pseudo-Hermitian Hamiltonian [14,24] times $e^{-i\frac{\varphi }{2}}$, as follows:

(5) $$H_{\varphi }=e^{-i\frac{\varphi }{2}}\left(\begin{array}{cc} re^{i\theta } & u\\ v & re^{-i\theta } \end{array}\right),$$

where φ, *r*, *u*, *v*, and θ are real parameters, the parity operator $P=\left(\begin{array}{cc} 0 & 1\\ 1 & 0 \end{array}\right)$, and φ is a fixed parameter that links to the symmetry.

The eigenvalues of $H_{\varphi }$ are $\varepsilon_{\pm }=e^{-i\frac{\varphi }{2}}\left(r\cos\theta \pm \frac{\omega_{0}}{2}\right)$ with respect to the two eigenvectors $|\varepsilon_{\pm }\rangle$, respectively, where $\omega_{0}=e^{i\frac{\varphi }{2}}\left(\varepsilon_{+}-\varepsilon_{-}\right)=2\sqrt{uv-r^{2}\sin^{2}\theta }$ is the energy difference of $H_{PH}:=e^{i\frac{\varphi }{2}}H_{\varphi }$. The exceptional points (EPs) of $H_{\varphi }$ in the parametric space [75] are composed of the points leading $\omega_{0}$ to be zero. The EPs of $H_{\varphi }$, forming the boundary of real ($\omega_{0}^{2}>0$) and imaginary ($\omega_{0}^{2}<0$) phases of $H_{PH}$, are also EPs of $H_{\varphi }$.

Investigations into novel phenomena using a controllable, currently available quantum device is one of the main tasks of quantum simulation. Because of the Hermiticity, the time-evolution of a Hermitian system is unitary and can be simulated by a conventional quantum system directly in the Hilbert space of the same dimensions.

We will simulate the two-dimensional time-evolutionary operator

(6) $$e^{-i\frac{t}{\hbar }H_{\varphi }},$$

which is not unitary. Therefore, instead of the Hermitian case using one qubit, we will construct a general *P*-PH-φ subsystem in a larger Hilbert space and simulate the time-evolution in the scheme of duality quantum computing using the LCU method.

Our quantum simulation method is applicable to the whole parametric space (except the EPs), including the neighborhoods of EPs. We still use the Hilbert–Schmidt inner product of the conventional quantum mechanics because this NH system will be simulated in a Hermitian system, while the pseudo-inner product introduced by A. Mostafazadeh [34] is well defined in this system.

### 4.2. UE of the Time-Evolutionary Operator

First of all, the UE techniques [8] will be applied to the non-unitary time-evolutionary operator in Equation (4). We calculate the UE terms of $e^{-i\frac{t}{\hbar }H_{\varphi }}$ in detail, which can be expanded by four or three UE terms in general, as follows:

(7) $$e^{-i\frac{t}{\hbar }H_{\varphi }}=f_{0}\sigma_{0}+f_{1}\sigma_{1}+f_{2}\left(i\sigma_{2}\right)+f_{3}\sigma_{3},$$

where the Pauli matrices $\sigma_{0}=\left[\begin{array}{cc} 1 & 0\\ 0 & 1 \end{array}\right]$, $\sigma_{1}=\left[\begin{array}{cc} 0 & 1\\ 1 & 0 \end{array}\right]$, $\sigma_{2}=\left[\begin{array}{cc} 0 & -i\\ i & 0 \end{array}\right]$, and $\sigma_{3}=\left[\begin{array}{cc} 1 & 0\\ 0 & -1 \end{array}\right]$, and $f_{k}=\left|f_{k}\right|e^{i\theta_{k}}$ (k=0,1,2,3) are the UE parameters, being time-dependent complex-functions of $H_{\varphi }$ in Equation (5), with no limitations on their norms. The explicit forms of $f_{k}$’s (k=0,1,2,3) are shown in Appendix A (i). To simplify the simulation, the number of UE terms should be further reduced. Thus, we merge the four UE terms in Equation (7) to three, as

(8) $$e^{-i\frac{t}{\hbar }H_{\varphi }}=e^{i\theta_{0}}\left|f_{0}\right|\sigma_{0}+e^{i\theta_{3}}\left|g_{1}\right|V_{1}+e^{i\theta_{3}}\left|g_{2}\right|V_{2}.$$

where the UE parameters $g_{1}$ and $g_{2}$ are complex functions of $f_{k}$’s (k=0,1,2,3) in Equation (7). The explicit forms of $g_{k}$ and $V_{k}$ (k=1,2) are presented in Appendix A (ii) and (iii), respectively.

The above UEs are valid for a general *P*-PH-φ Hamiltonian. If $f_{k}$’s (k=0,1,2,3) in Equation (7) satisfy one of the phase-matching conditions, which have been introduced and investigated in detail in [8] to judge the minimum numbers of terms in LCU for an arbitrary two-dimensional nonunitary operator, the UE terms in Equation (8) can be merged to fewer terms, as follows:

(9) $$e^{-i\frac{t}{\hbar }H}=h_{0}W_{0}+h_{1}W_{1}.$$

The explicit forms of $h_{0}$, $h_{1}$, $W_{0}$, and $W_{1}$ vary in different phase-matching conditions [8], and they are complex functions with respect to time *t*.

For convenience, we set a normalizing factor *f* as

(10) $$f=\left|f\right|=\sum_{k=0}^{3}\sqrt{{\left|f_{k}\right|}^{2}}=\sqrt{{\left|f_{0}\right|}^{2}+{\left|g_{1}\right|}^{2}+{\left|g_{2}\right|}^{2}}=\sqrt{{\left|h_{0}\right|}^{2}+{\left|h_{1}\right|}^{2}}.$$

Now, we are able to simulate the non-unitary evolution in Equation (6) using LCU in the scheme of duality quantum computing. A qudit- or a qudit–qubit-hybrid device is able to achieve the simulation, and qudits take advantage over qubits in some quantum algorithms [76] (e.g., they reach a higher accuracy when solving the eigenvalue problem using quantum phase estimation algorithms with qudits than with qubits [77]). However, we focus on quantum simulations using qubits here, since qubit-quantum computers are currently available technologies.

### 4.3. Qubit Simulation

Three qubits or fewer are able to simulate the time-evolution of a *P*-PH-φ system by our theory. In a general case, the three qubits are divided into an evolutionary qubit *e* and an ancillary subsystem *a* of the remaining two qubits. The evolutionary qubit will evolve as per Equation (6) with the assistance of the ancillary subsystem in a probabilistic way. Only a six-dimensional Hilbert subspace, extended by ${|00\rangle }_{a}{|k\rangle }_{e}$, ${|01\rangle }_{a}{k\rangle }_{e}$, and ${|10\rangle }_{a}{k\rangle }_{e}$ (k=0,1), is needed, while the remaining two dimensions are spared. The success probability using six dimensions is larger than that using the full eight dimensions [8].

**Figure 3:**
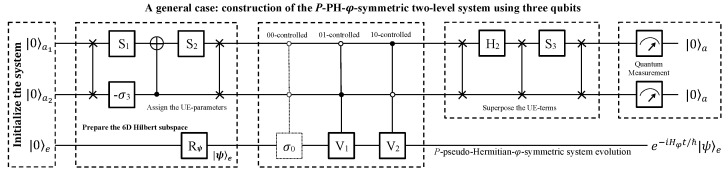
Three-qubit quantum circuit. The system is initialized to ${|00\rangle }_{a}{|0\rangle }_{e}$, and the evolutionary qubit *e* can be rotated to ${|\psi \rangle }_{e}$ by $R_{\psi }$ as needed. In the first block, operations prepare the six-dimensional subspace, and assign the three UE parameters. In the second block, three controlled-controlled operators (the first dashed one can be removed) generate the UE terms. In the third block, operations are applied on the ancillary system to superpose the three UE terms in Equation (8). Finally, quantum measurements are performed on the ancillary system to evolve the qubit *e* as the *P*-PH-φ system in an indeterministic way if ${|00\rangle }_{a}$ is output.

The quantum circuit used to achieve the quantum simulation is shown in Figure 3. At the beginning, the whole system is initialized to a pure state ${|00\rangle }_{a}{|0\rangle }_{e}$, and the evolutionary qubit *e* will be prepared in an arbitrary state ${|\psi \rangle }_{e}$, as needed by a single-qubit rotation $R_{\psi }$. The two ancillary qubits will assist the evolutionary qubit to evolve, governed by the *P*-PH-φ Hamiltonian. The first block aims at, on the one hand, deleting the basis ${|11\rangle }_{a}$ of the ancillary subsystem, so that the rest of the bases ${|00\rangle }_{a}$, ${|01\rangle }_{a}$ and ${|10\rangle }_{a}$ together with the two bases ${|0\rangle }_{e}$ and ${|1\rangle }_{e}$ of the evolutionary qubit are used to construct a six-dimensional subspace; on the other hand, it aims at assigning the three UE parameters in Equation (8) to ${|00\rangle }_{a}{|\psi \rangle }_{e}$, ${|01\rangle }_{a}{|\psi \rangle }_{e}$, and ${|10\rangle }_{a}{|\psi \rangle }_{e}$. This is the first key step to simulating the time-evolution. In detail, the first and second ancillary qubits are swapped, and two single-qubit rotations $S_{1}$ and $-\sigma_{3}$ are applied to them. Then, a controlled-NOT gate is applied, in which the first and the second qubits take roles as the target and control qubit, respectively. After the first qubit is rotated by $S_{2}$, the two ancillary qubits are swapped again. The explicit forms of two single-qubit operators are

(11) $$S_{1}=\frac{1}{f}\left[\begin{array}{cc} g_{1} & -\sqrt{{\left|f\right|}^{2}-{\left|g_{1}\right|}^{2}}\\ \sqrt{{\left|f\right|}^{2}-{\left|g_{1}\right|}^{2}} & g_{1}^{*} \end{array}\right]$$

and

(12) $$S_{2}=\frac{1}{\sqrt{{\left|f\right|}^{2}-{\left|g_{1}\right|}^{2}}}\left[\begin{array}{cc} g_{2}^{*} & f_{0}\\ -f_{0}^{*} & g_{2} \end{array}\right],$$

where $f_{0}$, $g_{1}$, $g_{2}$, and *f* are as in Equations (8) and (10).

In the second block of Figure 3, the three UE terms in Equation (8) will be generated. Notice that the unit matrix $C_{00-\sigma_{0}}$ is a trivial operation that can be removed in practice. The other two jointly controlled gates are necessary, and their matrix forms are

(13) $$C_{01-V_{1}}=\left[\begin{array}{cccc} \sigma_{0} & 0 & 0 & 0\\ 0 & V_{1} & 0 & 0\\ 0 & 0 & \sigma_{0} & 0\\ 0 & 0 & 0 & \sigma_{0} \end{array}\right]$$

and

(14) $$C_{10-V_{2}}=\left[\begin{array}{cccc} \sigma_{0} & 0 & 0 & 0\\ 0 & \sigma_{0} & 0 & 0\\ 0 & 0 & V_{2} & 0\\ 0 & 0 & 0 & \sigma_{0} \end{array}\right],$$

respectively. For the explicit expressions of $V_{1}$ and $V_{2}$, refer to Equation (8) and Appendix A (iii). Now, the three UE terms are generated and entangled with the three bases of the ancillary subspace.

The third block aims at superposing the three UE terms by swapping the two ancillary qubits three times and applying $H_{2}$ and $S_{3}$ in between them, as shown in Figure 3, where

(15) $$S_{3}=\frac{1}{\sqrt{3}}\left[\begin{array}{cc} \sqrt{2} & 1\\ 1 & -\sqrt{2} \end{array}\right].$$

Now, the whole system evolves to a superposition state

(16) $$\frac{1}{\sqrt{3}f}\left[{|00\rangle }_{a}e^{-i\frac{t}{\hbar }H_{\varphi }}{|\psi \rangle }_{e}+f\sum_{k=01,10}{|k\rangle }_{a}{|s_{k}\rangle }_{e}\right],$$

where values for $|s_{k}\rangle_{e}$ are not given explicitly because the relevant terms will be discarded after quantum measurements. Notice that the three UE terms are superposed as the time-evolution in the first term relevant to ${|00\rangle }_{a}$.

Finally, quantum measurements are performed on the ancillary subsystem. If it outputs the state ${|00\rangle }_{a}$, the evolutionary qubit will evolve to $e^{-i\frac{t}{\hbar }H_{\varphi }}{|\psi \rangle }_{e}$, which is governed by the NH Hamiltonian in Equation (5), with a success probability of

(17) $$\frac{1}{3f^{2}}{}_{e}\langle \psi |e^{i\frac{t}{\hbar }H_{\varphi }^{†}}e^{-i\frac{t}{\hbar }H_{\varphi }}{|\psi \rangle }_{e}.$$

If one of the remaining two results of ${|01\rangle }_{a}$ or ${|01\rangle }_{a}$ is measured, the simulation will be terminated. The whole process will then be started over until ${|00\rangle }_{a}$ is obtained. Therefore, it is an indeterministic protocol to simulate the time-evolution of *P*-PH-φ two-level systems.

**Figure 4:**
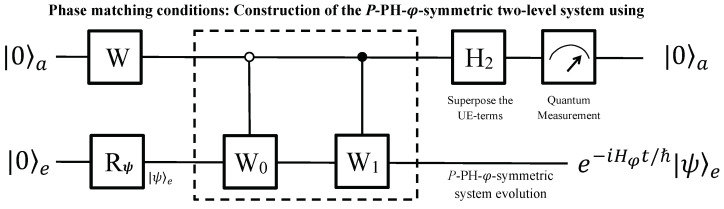
Two-qubit quantum circuit. The system includes an ancillary qubit *a* and an evolutionary qubit *e*, and is initialized to ${|0\rangle }_{a}{|0\rangle }_{e}$ at first. Then, the qubit *e* is rotated to ${|\psi \rangle }_{e}$ as needed by $R_{\psi }$. In the main part, operators are applied in series (i.e., a single-qubit rotation *W*, two controlled operators, and a Hadamard). Finally, the evolutionary qubit *e* will evolve as $e^{-i\frac{t}{\hbar }H_{\varphi }}{|\psi \rangle }_{e}$, if the ancillary qubit is measured in state ${|0\rangle }_{a}$.

The number of qubits can be reduced to two if the time-evolutionary operator in Equation (8) can be united into two UE terms, as in Equation (9), and the quantum circuit is shown in Figure 4.

The two-qubit system is initialized to ${|0\rangle }_{a}{|0\rangle }_{e}$, and then qubit *e* is rotated to ${|\psi \rangle }_{e}$ as needed. A single-qubit unitary

(18) $$W=\frac{1}{f}\left[\begin{array}{cc} h_{0} & -h_{1}^{*}\\ h_{1} & h_{0}^{*} \end{array}\right],$$

is applied on the ancillary qubit to assign the UE parameters. Notice that $h_{0}$ and $h_{1}$ always satisfy Equation (10), while their explicit forms change with different phase-matching conditions, as seen in [8].

Then, two controlled gates follow, which are

(19) $$C_{0-W_{0}}=\left[\begin{array}{cc} W_{0} & 0\\ 0 & \sigma_{0} \end{array}\right]$$

and

(20) $$C_{1-W_{1}}=\left[\begin{array}{cc} \sigma_{0} & 0\\ 0 & W_{1} \end{array}\right],$$

where the explicit forms of $W_{0}$ and $W_{1}$ are decided by the specific phase-matching conditions [8]. After a Hadamard $H_{2}$ is performed on the ancillary qubit, the two-qubit system evolves to

(21) $$\frac{1}{\sqrt{2}f}\left[{|0\rangle }_{a}e^{-i\frac{t}{\hbar }H_{\varphi }}{|\psi \rangle }_{e}+{|1\rangle }_{a}\left(h_{0}W_{0}-h_{1}W_{1}\right){|\psi \rangle }_{e}\right].$$

Similarly, the first term links to the time-evolution governed by the *P*-PH-φ Hamiltonian, while the second term will be discarded after quantum measurements.

Finally, a quantum measurement is performed on the ancillary qubit. If ${|0\rangle }_{a}$ is output, the evolutionary qubit *e* will evolve as $e^{-i\frac{t}{\hbar }H_{\varphi }}{|\psi \rangle }_{e}$ with a success probability of

(22) $$\frac{1}{2f^{2}}{}_{e}\langle \psi |e^{i\frac{t}{\hbar }H_{\varphi }^{†}}e^{-i\frac{t}{\hbar }H_{\varphi }}{|\psi \rangle }_{e}.$$

This is decided by not only by the initial state but also $H_{\varphi }$. If the ancillary qubit is observed in state ${|1\rangle }_{a}$, the process will be terminated and the result will be discarded. We start the quantum simulation again, and it continues until ${|0\rangle }_{a}$ is measured. From Equations (17) and (22), it can be seen that the success probability using two qubits is increased further than that using three qubits. Therefore, it is valuable to merge the UE terms before quantum simulation to increase efficiency. On one hand, this will save qubits, decreasing the complexities of the quantum circuit. On the other hand, it will enlarge the probability of simulating the *P*-PH-φ NH system successfully.

## 5. Experimental Proposals

Given that LCU and duality quantum algorithms have recently been successfully applied to similar types of quantum simulations in different experimental systems [23,25,26,27], we look forward to the implementation of available quantum devices. Since qubit-quantum devices have become available technologies, it is appropriate to simulate the pseudo-Hermitian-φ-symmetric systems using qubits for experimental implementations. Candidate qubit-systems include nuclear-magnetic-resonance (NMR) quantum simulators, quantum optics systems, superconductor quantum systems, two energy levels of ultracold atoms, and ion-trap systems. The operations in the quantum circuit can be realized by the related controlling methods and techniques.

Take an NMR quantum simulator as an example. The evolutionary and ancillary qubits are realized by the nuclei of spin-1/2. To initialize the pseudo-pure state, the spatial-averaging method [78] can be applied at the beginning of the experiment, and then a series of magnetic pulse sequences will be applied to realize the quantum gates in the quantum circuit. In detail, hard pulses are used to realize single-qubit rotations directly, while free evolutions of the two nuclei of spin-$\frac{1}{2}$ in a period [23] are necessary to realize controlled two-qubit gates.

By quantum optics, two orthogonal polarized directions of a photon take the role of a qubit. Single-qubit gates can be realized by a series of quarter-wave and half-wave plates [79]. The efficiency is too low in practice to realize a two-polarization-qubit gate, though the task can be achieved using measurement-induced nonlinearity [80]. A more practical method is to improve efficiency with the assistance of the degrees of freedom of photon locations utilized as the qubit basis (i.e., the location qubit), which can be prepared and operated by beam-splitters and Mach–Zehnder interferometers [81].

In addition, quantum processors (such as the IBM QE 5-qubit [82]) can be used to realize the quantum simulation in this work.

## 6. Conclusions

We complexly generalize the conventional pseudo-Hermitian system to a pseudo-Hermitian-φ-symmetric system by adding an extra freedom of symmetry to the original. Therefore, the pseudo-Hermitian system and its anti-symmetric counterpart can be seen as two special cases when the phase-angles φ are set to be 2kπ and (2k+1)π (*k* is integral), respectively. This can be analogous to the case of the PT, anti-PT, and PT-arbitrary-phase symmetries, or that of bosons, fermions, and anyons. We believe that more novel properties can be found in the generalized systems, and the conventional PH systems can be better investigated through extended freedom of symmetry (as we can understand a two-dimensional plane better when we are in a three-dimensional space). The well-defined inner product and pseudo-inner product are still valid for the PH-φ systems.

We mainly investigate quantum simulation of the PH-φ system. We show a schematic proposal for a general system using LCU in the scheme of duality quantum computing, and we propose in detail a general *P*-PH-φ two-level system. A minimum six-dimensional Hilbert space is necessary to simulate the time-evolution of an arbitrary *P*-PH-φ two-level system by our unitary expansion techniques, while four-dimensional Hilbert space is enough in some special cases. The simulation is achieved in an indeterministic way, and the success probability is decided by the initial state, the Hamiltonian, and the dimensions of the used Hilbert space. The fewer dimensions there are, the higher the success probability is. Therefore, it is meaningful to merge the UE terms based on our UE techniques and phase-matching conditions to reduce the dimensions required before quantum simulation. With this, the qubit source will be saved, and the success probability will be increased.

Finally, we discuss experimental implementations of available quantum devices, such as NMR, quantum optics systems, and IBM QE. Given that LCU and duality quantum algorithms have been recently successfully applied to similar types of quantum simulations experimentally, quantum simulation of a general *P*-PH-φ two-level system can soon be implemented on small quantum devices.

## Data Availability

Not applicable.

## Abbreviations

The following abbreviations are used in this manuscript:

NH	non-Hermitian
PT	parity-time-reversal
PH	pseudo-Hermitian
PHA	pseudo-Hermitian anti-symmetric
PH-φ	pseudo-Hermitian-φ-symmetric
*P*	parity
EP	exceptional point
LCU	linear combination of unitaries
NMR	nuclear magnetic resonance

## Appendix A

(i) Explicit forms of $f_{k}$’s (k=0,1,2,3):

(A1) $$f_{0}=m\left[a_{0}b_{0}-a_{2}b_{2}+i\left(-a_{1}b_{1}+a_{3}b_{3}\right)\right],$$

(A2) $$f_{1}=m\left[\left(a_{0}b_{1}-a_{2}b_{3}+a_{3}b_{2}\right)-ia_{1}b_{0}\right],$$

(A3) $$f_{2}=m\left[\left(a_{0}b_{2}+a_{3}b_{1}-a_{1}b_{3}\right)-ia_{2}b_{0}\right]$$

and

(A4) $$f_{3}=m\left[a_{3}b_{0}+i\left(a_{1}b_{2}-a_{2}b_{1}+a_{0}b_{3}\right)\right],\;\mathrm{where}$$

(A5) $$m=e^{-i\frac{t}{\hbar }re^{\left(-\varphi /2\right)}\cos\theta },$$

(A6) $$a_{0}=\cos\alpha =\cos\left(\omega_{a}t/2\hbar \right),$$

(A7) $$a_{1}=\left(u+v\right)\cos\left(\varphi /2\right)\sin\alpha /\omega_{a},$$

(A8) $$a_{2}=\left(u-v\right)\cos\left(\varphi /2\right)\sin\alpha /\omega_{a},$$

(A9) $$a_{3}=2r\cos\left(\varphi /2\right)\sin\theta \sin\alpha /\omega_{a},$$

(A10) $$\omega_{a}=2\left|\cos(\varphi /2)\right|\sqrt{uv-r^{2}\sin^{2}\theta };$$

and

(A11) $$b_{0}=\cos\beta =\cos\left(\omega_{b}t/2\hbar \right),$$

(A12) $$b_{1}=-\left(u+v\right)\sin\left(\varphi /2\right)\sin\beta /\omega_{b},$$

(A13) $$b_{2}=-\left(u-v\right)\sin\left(\varphi /2\right)\sin\beta /\omega_{b},$$

(A14) $$b_{3}=-2r\sin\left(\varphi /2\right)\sin\theta \sin\beta /\omega_{b},$$

(A15) $$\omega_{b}=2\left|\sin(\varphi /2)\right|\sqrt{r^{2}\sin^{2}\theta -uv}.$$

(ii) Explicit forms of $g_{1}$ and $g_{2}$:

$g_{1}$ and $g_{2}$ are obtained from $f_{k}=\left|f_{k}\right|e^{i\theta_{k}}$ (k=0,1,2,3), that is,

(A16) $$g_{1}=e^{i\theta_{3}}\sqrt{{\left|f_{3}\right|}^{2}+{\left|f_{1}\right|}^{2}\cos^{2}\varphi_{1}+{\left|f_{2}\right|}^{2}\sin^{2}\varphi_{2}}$$

and

(A17) $$g_{2}=e^{i\theta_{3}}\sqrt{{\left|f_{1}\right|}^{2}\sin^{2}\varphi_{1}+{\left|f_{2}\right|}^{2}\cos^{2}\varphi_{2}},$$

where

(A18) $$\varphi_{1}=\theta_{1}-\theta_{3}\;\mathrm{and}\;\varphi_{2}=\theta_{2}-\theta_{3}.$$

(iii) Explicit forms of $V_{1}$ and $V_{2}$:

(A19) $$\left[\begin{array}{cc} \cos\zeta_{1} & e^{i\phi_{1}}\sin\zeta_{1}\\ e^{-i\phi_{1}}\sin\zeta_{1} & -\cos\zeta_{1} \end{array}\right]\;\mathrm{and}\;\left[\begin{array}{cc} 0 & e^{i\phi_{2}}\\ -e^{-i\phi_{2}} & 0 \end{array}\right],$$

where $\phi_{1}$ is decided by

(A20) $$\cos\phi_{1}=\frac{\left|f_{1}\right|\cos\varphi_{1}}{\sqrt{{\left|f_{1}\right|}^{2}\cos^{2}\varphi_{1}+{\left|f_{2}\right|}^{2}\sin^{2}\varphi_{2}}}$$

and

(A21) $$\sin\phi_{1}=\frac{\left|f_{2}\right|\sin\varphi_{2}}{\sqrt{{\left|f_{1}\right|}^{2}\cos^{2}\varphi_{1}+{\left|f_{2}\right|}^{2}\sin^{2}\varphi_{2}}};$$

and $\phi_{2}$ is decided by

(A22) $$\cos\phi_{2}=\frac{\left|f_{2}\right|\cos\varphi_{2}}{\left|g_{2}\right|}\;\mathrm{and}\;\sin\phi_{2}=\frac{\left|f_{1}\right|\sin\varphi_{1}}{\left|g_{2}\right|};$$

$\zeta_{1}$ is decided by

(A23) $$\cos\zeta_{1}=\frac{\left|f_{3}\right|}{\left|g_{1}\right|}\;\mathrm{and}\;\sin\zeta_{1}=\frac{\sqrt{{\left|f_{1}\right|}^{2}\cos^{2}\varphi_{1}+{\left|f_{2}\right|}^{2}\sin^{2}\varphi_{2}}}{\left|g_{1}\right|}.$$
